# Anesthesia protocol for ear surgery in Wistar rats (animal research)

**DOI:** 10.1002/ame2.12198

**Published:** 2022-01-18

**Authors:** Abhijeet Bhatia, Pranjal Pratim Saikia, Barilin Dkhar, Haphidasara Pyngrope

**Affiliations:** ^1^ Department of ENT North Eastern Indira Gandhi Regional Institute of Health & Medical Sciences (NEIGRIHMS) Shillong India; ^2^ Department of Pharmacology (Animal House) North Eastern Indira Gandhi Regional Institute of Health & Medical Sciences (NEIGRIHMS) Shillong India

**Keywords:** isoflurane, ketamine, otology, Wistar rat, xylazine

## Abstract

**Objective:**

To formulate an anesthesia protocol for safe and satisfactory anesthesia for ear surgery in rats.

**Methods:**

The rats were anesthetized with xylazine (10 mg/kg body weight) and ketamine at doses of 80, 50, 40, and 30 mg/kg body weight or with isoflurane anesthesia (2%–3.5% in 100% oxygen; maintenance dose 1.5%–3.5%). The anesthesia induction, surgery, and recovery time were recorded.

**Results:**

In total, 17 rats were induced by varying doses of ketamine‐xylazine and 28 rats with isoflurane. Mean induction time with ketamine‐xylazine was 6 ± 2.9 min compared with 3.8 ± 1.1 min with isoflurane. Mean recovery time with ketamine‐xylazine was 142.6 ± 49.3 min compared with 4.1 ± 1.2 min with isoflurane. A mortality of 4 animals after developing dyspnea was recorded with ketamine‐xylazine.

**Conclusion:**

Isoflurane anesthesia offers appropriate induction and recovery times and low mortality rates for the surgeries performed. Isoflurane anesthesia offers reliable results for ear surgery in rats. However, more equipment and technical skills are needed.

## 1 INTRODUCTION

Anesthetic agents that are safe, repeatable, inexpensive, and minimally traumatic are ideal for invasive animal experiments. Ketamine hydrochloride is one of the most commonly used injectable anesthetic agents, and its usefulness as a rapidly acting general anesthetic has been confirmed. Xylazine hydrochloride is an effective sedative and commonly used as an adjunct to anesthesia in animals. A combination of ketamine and xylazine is commonly used to anesthetize rats, mice, and other rodents.^1^ This combination is known to have a number of advantages as opposed to using either drug alone. Xylazine supplements ketamine with its analgesic properties, muscle relaxation, and sedation, which are beneficial in reducing side effects of ketamine such as tremor and muscle rigidity. Moreover, the combination of ketamine and xylazine provides an optimal anesthesia without the need for specialized equipment.^2^

Among the volatile anesthetic agents, isoflurane is a commonly used inhalation anesthetic in animal research, known for its rapid induction and recovery, and control over depth of anesthesia. It has been reported to undergo less biotransformation than other agents, thus making it a suitable choice for in vivo studies. It is commonly used for short‐ and moderate‐duration procedures. Inhalation anesthesia, however, requires substantial physical infrastructure and trained manpower.^2^, ^3^, ^4^

Ear surgeries in rats for otological research are usually short‐to‐moderate‐duration procedures performed using an operating microscope. Such procedures present unique challenges for the anesthetist. The surgical field is cramped due to close proximity to the other sense organs and to the airway that needs to be accessible to the anesthetist. Such unique challenges will dictate the choice of the anesthesia technique and agents. Though extensive studies exist on the merits and demerits of the various anesthesia techniques and agents used in the current study, they have not been studied in the context of the otology procedures. The present work describes the experience of the authors while establishing the protocols for injectable and inhalational anesthesia in adult Wistar rats for nonmutilating, short‐to‐moderate‐duration survival ear surgeries by post‐aural approach.

## 2 MATERIALS AND METHODS

This prospective cohort study was conducted on clinically healthy adult Wistar rats (*Rattus norvegicus*) in the animal house of North Eastern Indira Gandhi Regional Institute of Health and Medical Sciences, Shillong, India (NEIGRIHMS). The study was carried out after approval by the Institutional Animal Ethics Committee (letter no. NEIGR/Pharma‐AH/IAEC/2016/07, dated November 8, 2017) in accordance with the guidelines of the Committee for the Purpose of Control and Supervision of Experiments on Animals (CPCSEA), India. All the animals (Saha Enterprises, Kolkata, India) were maintained in polycarbonate cage (BIK Industries, India) with corn cob bedding (qCob, Altromin, Germany) under laboratory conditions of temperature 22 ± 2°C, relative humidity 45 ± 5%, and 12 h light cycle and were allowed food (standard cereal‐based pellet diet) (Altromin, Germany) and water ad libitum.

### 2.1 Experimental design

This study was conducted as a part of an ongoing project that involved ear surgery in rats, as described later. Only clinically healthy animals were selected for the study. The animals had been housed in the Animal House for at least 2 months before they were operated. Rats that had been operated previously for any other disease were excluded.

The rats procured for the study were shortlisted and numbered sequentially. The rats for each procedure were identified 1 day prior in no particular order after considering the general clinical health of the rats. The observations were recorded with the veterinary anesthetist blinded to the outcomes. The veterinary anesthetist (P.P.S.) and surgeon (A.B.) were blinded to the numbering sequence, identification of rats for surgery, and observations. The data were analyzed by A.B. The results were noted with reference to otological surgeries conducted in this study.

The rats were fasted for 3 h and then anesthetized with either intramuscular injections of ketamine (Ketamax, Troikaa Pharmaceuticals Ltd., India) (30–80 mg/kg bodyweight) and xylazine (Xylaxin, Indian Immunologicals Limited, India) (10 mg/kg bodyweight) or isoflurane (Aerrane, Baxter Healthcare Corporation, USA) by inhalation (2.5%–3% induction, then 2%–2.5% maintenance, with oxygen) at the rate of 1.5 L/min. Once anesthetized, carboxymethyl cellulose sodium ophthalmic eye drops (NatTears Lite, Kaizen Pharmaceuticals, India) were instilled in the eyes. The body heat during anesthesia was maintained at 38°C with a regulated heating pad (HBLD‐01, Orchid Scientific & Innovative India Pvt. Ltd, Nasik, India) till recovery from anesthesia. All animals were used for surgical implantation of a biocompatible polyurethane strip in post‐aural region in the subcutaneous plane. Some animals were also subjected to exploration of the middle ear before implantation in the subcutaneous plane. None of the animals was euthanized. All anesthesia‐related procedures and surgeries were performed by a single veterinarian and surgeon, respectively. In all the animals, anesthesia induction time was recorded as the time from application of the anesthetic agents to loss of righting and pedal reflexes in forelimbs and hind limbs and corneal reflex. Recovery time was measured as the time from the administration of last dose of injectable anesthetic agent until complete recovery of righting reflex. In case of inhalational anesthesia, the recovery time was measured from the time of withdrawal of anesthetic agent to recovery of righting reflex. The duration for which the reflexes remained suspended was the anesthesia time. Surgery time was defined as the time from preparation of the surgical part to dressing. Pedal reflex was determined by firmly pinching the pad of a forelimb and hind limb with blunt forceps in a manner that would not cause injury. To assess the corneal reflex, air was feebly blown in the eye. Righting reflex was assessed by placing the animal supine or in lateral position and noting if the animal attempts to regain the normal position with all four feet on the ground. The depth of anesthesia was monitored by periodically assessing the pedal reflexes, body motion and responses to the surgical procedure. The respiratory rate, mucous membrane and rectal temperature were also monitored. Ketamine/xylazine anesthesia is used often in the authors' institute for studies on small animals. However, since the results in terms of recovery time and mortality were unacceptable for the procedures in the current study, the decision was made to switch to isoflurane anesthesia. The different anesthesia regimens administered are described below.

### 2.2 Ketamine/xylazine (injectable anesthesia)

Xylazine (10 mg/kg) followed by ketamine in variable doses (80, 50, 40, and 30 mg/kg) was injected intramuscularly into the femoral musculature of the hind leg. After loss of the reflexes, the rat was placed in prone position on the heating pad on an operating table. Following the surgical procedure and wound dressing, the rat was kept under observation till the righting reflex returned. Thereafter, the animal was placed back in its cage and kept in the animal housing room under controlled environmental conditions.

### 2.3 Isoflurane

The rat was placed in an induction chamber and subjected to induction dose varying from 2% to 3.5% isoflurane in 100% oxygen at a rate of 1.5 L/min till loss of the reflexes. The rat was then placed in prone position on the heating pad, and then the dose of isoflurane was individually regulated at a maintenance dose also varying from 1.5% to 3.5% administered through a sterile nose cone (NCON‐D; Orchid Scientific & Innovative India Pvt. Ltd, Nasik, India). A gaseous anesthesia system (Hospital Devices, India) was used for this purpose. Depth of anesthesia was monitored regularly. After surgery and wound dressing, isoflurane administration was ceased and the rat was administered oxygen and monitored till its righting reflex returned.

The animals were administered a single dose each of ceftriaxone (25–50 mg/kg, intramuscular [i.m.]) and meloxicam (10 mg/kg, subcutaneous [s.c.]) intraoperatively. Postoperative care included once daily administration of ceftriaxone and meloxicam for a period of 3–5 d and daily wound dressing for 7 d.

### 2.4 Statistical analysis

All statistical analyses were performed using GNU PSPP version 1.2.0‐g0fb4db, software. The differences in the anesthesia induction time and recovery time between ketamine‐xylazine and isoflurane anesthetized groups were analyzed statistically using independent samples *t*‐test. A *p* value less than .05 was considered statistically significant.

## 3 RESULTS

A total of 45 animals (27 male; 17 female) with gender of one rat unrecorded, 4–18 months old (mean 8.7 ± 3.8 months) and weighing 100–570 g (mean 252.9 ± 107.1 g) were included in the surgery. Seventeen rats also underwent middle ear exploration, prior to implant insertion. Surgery time on such animals was longer than on the rest of the animals.

### 3.1 Injectable anesthesia

Initially, 4 rats (4 ears) were administered 80 mg/kg ketamine and 10 mg xylazine. The anesthesia induction time was in the range of 4–6 min (mean 5 ± 1 min), and the recovery time was in the range of 125–165 min (mean 146.7 ± 20.2 min) against a mean surgery time of 20 ± 20 min (Table 1). One animal developed dyspnea during recovery from anesthesia and died. The recovery time from this animal was, therefore, not recorded.

**TABLE 1:** Anesthesia time for ketamine/xylazine and isoflurane anesthesia

	Ketamine/xylazine (*n* = 17) [mean ± standard deviation (SD)]					Isoflurane (*n* = 28) (mean ± SD)
	80 mg/kg KX (*n* = 4)	50 mg/kg KX (*n* = 9)	40 mg/kg KX (*n* = 2)	30 mg/kg KX (*n* = 2)	Overall KX (*n* = 17)	
Anesthesia induction time (min)	5 ± 1	5.4 ± 2.6	10.5 ± 3.5	5.5 ± 2.1	6 ± 2.9	3.8 ± 1.1
Surgery duration (min)	30 ± 20	17.8 ± 12.2	7.5 ± 2.1	116 ± 2	31 ± 35.3	29.8 ± 28.9
Recovery time (min)	146.7 ± 20.2	155 ± 67.1	122 ± 3.5	113 ± 9.9	142.6 ± 49.3	4.1 ± 1.2

Due to the long recovery period with 80 mg/kg ketamine, the dose of ketamine was reduced to 50 mg/kg. Nine animals were anesthetized with this reduced dosage. The induction time was in the range of 2–11 min (mean 5.4 ± 2.6 min), while the time taken by the animals to recover from anesthesia was in the range of 70–280 min (mean 155 ± 67.1 min). The mean time taken for the surgical procedure was 17.8 ± 12.2 (Table 1). Two animals died after developing dyspnea during the recovery period.

Since the recovery time continued to be unacceptably high, ketamine dose was further reduced to 40 mg/kg. Two animals were administered this reduced dose. The induction time was 8 and 13 min (mean 10.5 ± 3.5 min). The recovery time was 120 and 125 min (mean 122.5 ± 3.5 min) against surgery times of 6 and 9 min (mean 7.5 ± 2.1 min). No animal died with this dosage regimen, though one animal developed dyspnea during the postoperative recovery.

In a continued attempt to optimize the ketamine dose in view of the prolonged recovery time, 30 mg/kg ketamine was given in 2 animals. The induction times were 4 and 7 min (mean 5.5 ± 2.1 min), and recovery times were 106 and 120 min (mean 151.5 ± 55.5 min). The surgical times were 101 and 131 min (mean 116 ± 21.2 min). Postoperatively, one animal displayed signs of severe pain, and hence the antibiotic and analgesic regimen was extended to 5–7 d. One animal died after developing dyspnea during the recovery period.

Therefore, a total of 17 animals underwent the procedures using ketamine‐xylazine anesthesia. A total of 4 rats died in the immediate postoperative period, of which 1 was administered 80 mg/kg ketamine, 2 were administered 50 mg/kg ketamine, and 1 was administered 30 mg/kg ketamine; all 4 deaths were due to dyspnea. None of the animals included in the study required top‐up doses. During the postoperative period, no local complications were observed following i.m. injection of the anesthetic agent. The induction time in the rats anesthetized with ketamine/xylazine was 6 ± 2.9 min, and the recovery time was 142.6 ± 49.3 min against a mean surgery time of 31 ± 35.3 min.

During the process of administering injectable anesthesia, it was observed that the process of induction and maintenance of anesthesia required minimal equipment. Access to the surgical field, that is, the periauricular region, was unhindered. Manipulation of the head and body during the surgical procedure was also unhindered.

### 3.2 Gaseous anesthesia

Twenty‐eight animals were anesthetized with a mean induction dose of isoflurane of 2.9 ± 3% isoflurane and 1.5 L/min oxygen in the induction chamber. The mean maintenance dose of isoflurane was 2.5 ± 0.5%. During the surgical procedure, 10 animals displayed dyspnea; however, this complication was managed uneventfully by reducing the maintenance dose of isoflurane to 1.5% and increasing the oxygen volume to 2–2.5 L/min. The time taken to induce anesthesia was 2–8 min (mean 3.8 ± 1.1 min). The mean time taken to complete the surgical procedure was 29.8 ± 28.9 min, while the time taken by the animals to recover from anesthesia was 1–6 min (mean 4.1 ± 1.2 min) (Table 1). However, the recovery time was recorded in only 14 animals. No mortality was recorded, and all the animals recovered uneventfully. Almost all the animals anesthetized with ketamine‐xylazine and isoflurane displayed passive behavior for 3–5 d after surgery. The health conditions of the animals gradually improved, and the animals were active and healthy 7–10 d after surgery. The induction time and recovery time with injectable anesthesia were both significantly greater than that with inhalational anesthesia (*p* = .001 and *p* < .001, respectively, with equal variances assumed).

The procedure for administration of inhalational anesthesia was observed to be technically demanding, with use of substantial equipment. The anesthesia was administered through a nose cone, which significantly hindered access to the surgical field. The manipulation of the head and body was also cumbersome. However, it was also observed that with experience, and a short learning curve, the issues of access to the surgical field and convenience of manipulation were circumvented.

## 4 DISCUSSION

Though the induction time with isoflurane anesthesia was significantly less than that with ketamine‐xylazine, it was of an acceptable duration with both agents. However, the recovery time was unacceptably prolonged with all the tested doses of ketamine‐xylazine with respect to the surgery time. Further, a mortality of 5 animals was also recorded, following dyspnea in animals anesthetized with ketamine‐xylazine. The recovery time with isoflurane was of an acceptable duration with respect to surgery time and significantly less than that with ketamine‐xylazine. Although complications such as dyspnea were encountered intraoperatively in 3 animals, recovery was uneventful because of greater control over anesthesia depth. There were no cases of mortality or sequelae recorded in isoflurane‐anesthetized animals.

Ketamine is an *N*‐methyl‐d‐aspartate receptor antagonist, classified as a dissociative anesthetic that produces analgesia and immobility.^1^ Xylazine is an α2 adrenoreceptor agonist.^1^ It acts as a sedative, analgesic, and muscle relaxant. The properties of ketamine and xylazine complement each other. The sedative and muscle‐relaxant properties of xylazine are beneficial in reducing side effects of ketamine such as tremor and muscle rigidity. Ketamine‐xylazine is a commonly used and cost‐effective injectable anesthetic combination for rodents, particularly for long‐duration procedures.^1^, ^5^ Though ketamine‐xylazine can be administered by different routes, the intramuscular route is less stressful for the animals, and also avoids inadvertent injection into the gastrointestinal tract.^6^ In rats, the half‐lives of ketamine and xylazine are approximately 2 and 1 h, respectively, and both drugs are chiefly metabolized in the liver.^7^ For anesthesia duration of 60–80 min, the doses of ketamine and xylazine recommended by various laboratories in United States range from 40 to 100 mg/kg and 5 to 13 mg/kg body weight, respectively.^8^, ^9^ Ketamine and xylazine at the recommended doses have been reported in various studies to have an induction time ranging from 5 to 10 min and recovery period ranging from 60 to 120 min. The long recovery time might be attributed to its slow metabolism.^10^, ^11^, ^12^, ^13^ The induction time was similar to that in the current study, but the recovery time was shorter, though it was significantly longer than the surgery time required for ear surgeries conducted in the current study at all recommended doses. Depth of anesthesia was satisfactory for the surgical procedures performed, and the surgical field was unhindered with the combination. Repeated manipulation of the head required for the surgical procedures on the ear was unhindered as well.

Although the use of ketamine‐xylazine offers numerous advantages, the side effects associated with their use have also been reported to include respiratory depression, which might even be fatal. Hypothermia is a major risk in anesthetized animals, hence the need to maintain body temperature during the procedure. Muscle necrosis has frequently been observed following intramuscular administration of the combination. Cardiac depression has also been commonly observed.^2^, ^14^ In the current study, 5 rats developed dyspnea and could not recover from anesthesia. No abnormality was noted at the injection site in any of the animals after i.m. injection. In view of the safety concerns, injectable anesthetics, primarily ketamine‐xylazine, are preferred anesthetic agents in settings where infrastructure for inhalational anesthesia is not available.^2^, ^14^ Inhalation anesthesia using isoflurane is the most widely used method of general anesthesia in animal research as it produces rapid induction and recovery from anesthesia and undergoes less biotransformation.^15^ Other volatile inhalational anesthetics such as ether, halothane, methoxyflurane, isoflurane, sevoflurane, desflurane, and enflurane are also commonly used in different species. Isoflurane has been successfully used to anesthetize rats at a concentration of 2.5%–5% with a maintenance dose of 1%–3%.^16^, ^17^ The use of isoflurane in the current study enabled regulation of depth of anesthesia in real time. However, isoflurane has been reported to depress cardiovascular function in a dose‐dependent manner.^6^, ^18^ Isoflurane administration in the current study necessitated the use of a nose cone, which limited the manipulation of the head during the surgery, and also interfered with the surgical access. This shortcoming was overcome with experience. The nose cone was sterilized before use. Since inhalation anesthesia provided greater safety, appropriate induction and recovery times and real‐time regulation of depth of anesthesia, greater numbers of animals were operated using the technique, and injectable anesthesia was not used thereafter.

Ear surgery in rats is routinely conducted for numerous otological research studies. The ideal anesthetic agent and technique provides appropriate induction and recovery periods in comparison with the surgery time, along with adequate and controlled depth of anesthesia. The choice of anesthesia in such procedures is often crucial in determining the outcome of the surgery and the general well‐being of the animal. Middle ear surgeries are of moderate duration. They are usually survival, nonmutilating surgeries. In the current study, isoflurane anesthesia provided superior outcomes with respect to morbidity and mortality of the animals. It proved to be more reliable in terms of the ability to regulate the anesthesia duration for the required surgical period, and it induced a short recovery time with no mortality and minimal morbidity recorded. However, as described in the results section, it requires more equipment and, hence, a substantially higher cost. The inhalation anesthesia also involves technical expertise. The nose cone used for administration of inhalational anesthesia interferes with the surgical field and hinders manipulation of the head and body during the surgical procedures. However, it was also observed that these issues could be circumvented with a short learning curve. As a consequence, the constraints mentioned above ceased to be a decisive factor in the choice of anesthesia. In contrast, injectable anesthesia required minimal equipment, and less technical expertise. It also offered free, unhindered access to the surgical field and ease of manipulation during the procedures.

Thus, poor anesthesia outcomes for the otological procedures conducted with injectable anesthesia but low cost and technical ease were weighed against superior anesthesia outcomes but high cost and technically demanding procedures with inhalational anesthesia. The benefits offered by superior outcomes with inhalational anesthesia far outweighed the drawbacks listed above, and it was the obvious choice of anesthesia technique for the authors for moderate‐duration nonmutilating, survival surgeries on the middle ear of rats. The only factor that might affect the choice of anesthesia for such surgeries is the availability of equipment for inhalational anesthesia.

## 5 CONCLUSIONS

Isoflurane anesthesia provided superior outcomes with respect to morbidity and mortality of the animals. It proved to be more reliable in terms of the ability to regulate the anesthesia duration for the required surgical period, and it induced a short recovery time with no mortality and minimal morbidity recorded. However, it entails more equipment and, hence, a substantially higher cost. The inhalation anesthesia also involves technical expertise.

## CONFLICT OF INTEREST

The authors have no conflicts of interest to disclose.

## AUTHOR CONTRIBUTIONS

Dr Abhijeet Bhatia: Made substantial contributions to the conception and design of data, acquisition of data, and analysis and interpretation of data; revised the article critically for important intellectual content; approved the final version to be published. Dr Pranjal Saikia: Made substantial contributions to the acquisition, analysis, and interpretation of data; revised the article critically for important intellectual content; approved the final version to be published. Dr Barilin Dkhar: Made substantial contributions to acquisition and analysis of data; drafted the article. Ms Haphidasara Pyngrope: Made substantial contributions to the acquisition and analysis of data and drafting of article.
